# A Use Case for Generative AI in Medical Education

**DOI:** 10.2196/56117

**Published:** 2024-06-07

**Authors:** Tejas C Sekhar, Yash R Nayak, Emily A Abdoler

**Affiliations:** 1 Rush Medical College, Chicago, IL, United States; 2 University of Michigan Medical School, Ann Arbor, MI, United States

**Keywords:** medical education, med ed, generative artificial intelligence, artificial intelligence, GAI, AI, Anki, flashcard, undergraduate medical education, UME

 A recent study explored the novel application of generative artificial intelligence’s (GAI’s) capabilities with regard to Anki using a new methodology (“Anki Tagger”), leveraging OpenAI’s ChatGPT-3.5 to tag and stratify flashcards from the AnKing deck, which are most aligned with a medical school’s curriculum and involve a minimal cost and time expenditure [1]. To the best of our knowledge, their work represents the first publication demonstrating early proof of concept of GAI applied to Anki, a spaced repetition flashcard application designed to promote long-term retention of learned content. A major benefit of their approach is the ability to streamline and automate the otherwise time-consuming and resource-intensive process of manually comparing medical school curricula against the widely used and crowdsourced AnKing deck.

Medical students who use Anki may use decks prepared by more senior students at their medical school, the AnKing deck (a reputable and comprehensive set of >35,000 flashcards and growing daily, collaboratively maintained largely by current and graduated medical students), or a combination thereof. Research indicates that daily Anki use is associated with increased USMLE (United States Medical Licensing Examination) Step 1 scores and higher sleep quality—indicators of academic performance and personal well-being, respectively [2]. Given the prevalent usage and growing adoption of Anki among medical students, applications of GAI and large language models (LLMs) to Anki workflows may be beneficial. Even considering their present shortcomings, LLMs may provide a unique opportunity to significantly impact medical education in the intermediate term, especially given the propensity of contemporary medical students to supplement didactic learning with web-based learning resources [3].

Furthermore, LLMs with GAI capabilities, such as ChatGPT and Med-PaLM, have the potential to answer medically related questions [4] and—intriguingly for the medical education community—can pass the USMLE [5]. Such a notable feat by LLMs necessitates reevaluation of the future of medical training and practice while carefully considering the relevant ethical issues and current limitations of GAI, such as their susceptibility for generating misinformation through a process known as “hallucination.” As GAI and LLMs become increasingly integrated in daily practice, similar and iteratively improved methodologies represent a way for educators and learners alike to benefit considerably by better aligning flashcards from the comprehensive AnKing deck with in-house curricula in preparation for medical licensing examinations such as USMLE Step 1. Future applications of GAI in undergraduate medical education may involve the implementation of AI-assisted features directly built into preferred educational tools and resources, allowing students increased customization with options for multimodal output beyond solely text.

## Abbreviations

GAI: generative artificial intelligence
LLM: large language model
USMLE: United States Medical Licensing Examination
